# The thorax of *Eubleptus* illuminates evolutionary history of winged insects

**DOI:** 10.1016/j.isci.2026.116838

**Published:** 2026-07-29

**Authors:** Jakub Prokop, Klára Daňková, Louis Sampson, Pavel Sroka

**Affiliations:** 1 Department of Zoology, Faculty of Science, Charles University, Viničná 7, 128 00 Praha 2, Czech Republic; 2 Biology Centre of the Czech Academy of Sciences, Institute of Entomology, Branišovská 31, 37005 České Budějovice, Czech Republic

**Keywords:** insecta, palaeodictyoptera, pleuron, pterygota evolution, protopterygote, insect flight

## Abstract

The hexapod thorax represents a specialized tagma for body locomotion, and its formation was one of the crucial steps in arthropod evolutionary history. In insects, the thoracic morphology was crucial for the acquisition of wings, which was closely connected with the reinforcement of the lateral thoracic wall (pleuron). Here, we uncover the structure of the pleuron for the first time in a Carboniferous palaeodictyopteran, *Eubleptus danielsi* from the Mazon Creek Lagerstätte (Illinois, USA). We reconstructed a 3D model of *E. danielsi* and compared flight-related traits with a broad range of extant insect taxa. The thoracic pleuron is strikingly different across the main pterygote lineages. Whereas the pleura of Odonata and Ephemeroptera exhibit specialization with many autapomorphic characters, the arrangement observed in *E. danielsi* actually resembles the pattern seen in Neoptera. It is plausible that this represents a pattern close to that of the protopterygote, the hypothetical common ancestor of Pterygota.

## Highlights

• Carboniferous palaeodictyopteran with uniquely preserved lateral body wall
• First 3D reconstruction of thorax in one of the earliest insect fliers, 307 Mya
• Pleuron in *Eubleptus* is comparable to hypothetical pterygote ancestor
• Flight skills of *Eubleptus* compared to extant taxa, addressing evolution of flight

## Graphical abstract

**Figure:**
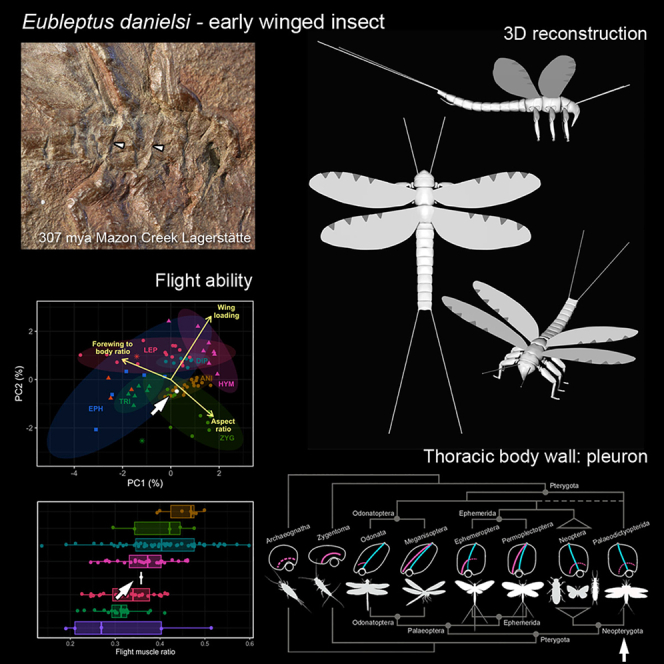

## Teaser

Zoology; Entomology; Evolutionary biology; Paleobiology

## Introduction

The hexapod thorax is the tagma specialized for locomotion, and its morphology has been studied from various aspects, particularly in connection to the evolution of flight. A number of studies deal with the comparative morphology of the wing base, lateral sclerites, and structure of the pterothoracic muscles among the main lineages of Pterygota to uncover the origin of insect flight, studying homologies, ordinal relationships, and reconstructing the ancestral state of characters.^1^^,^^2^^,^^3^^,^^4^^,^^5^^,^^6^^,^^7^^,^^8^ The part of the thorax that underwent the largest structural changes during the process of wing acquisition is represented by the reinforcement of the pleuron, the thoracic lateral body wall. However, this part of the thorax has rarely been studied in the context of fossil representatives.

The arrangement of the pleura is diverse in various groups of insects, and it is still unknown how the pleuron and overall morphology looked at the very beginning of winged insect evolution. Various authors have discussed what the hypothetical shared common ancestor, the “protopterygote” looked like; which presumably also depends on how the wings originally evolved. Unfortunately, the transition from apterygotes to flying winged insects is poorly documented in the fossil record^9^^,^^10^^,^^11^ and the process of definitive emergence of insect flight remains unknown. Molecular clock analyses give a rough emergence date of 400–405 Mya.^12^^,^^13^ The first arboreal environments may have emerged during the Silurian-Devonian Terrestrial Revolution.^14^ Directed aerial descent (DAD) may have developed during this time, enabling the protopterygote to go from parachuting from plant to plant to gliding between trees, and finally to flapping flight.

If the use of wings first emerged in an aquatic/semi-aquatic environment, as suggested by Wigglesworth,^15^ they may have been used to skim the surface.^16^^,^^17^^,^^18^ They may have played a role in respiration, as has been suggested in palaeodictyopterid nymphs.^19^ The articulated flaps could have been primarily for locomotion (skimming or swimming), and secondarily for respiration, or vice versa. In conclusion, the origin of wings is still debated, and the reconstruction of the protopterygote morphology is thus difficult.^20^ However, the studies focusing on ancient groups such as Palaeodictyopterida, and the morphology of their thorax, might provide us with useful clues.

While the thoracic morphology of extant insect orders is rather well documented, our knowledge on their extinct relatives is scarce.^21^^,^^22^^,^^23^^,^^24^ If we strive to reconstruct the ancestral structure of the pleuron in Pterygota, we must compare its arrangement in the three main phylogenetic lineages of the winged insects, the Ephemeroptera, Odonata, and Neoptera.^12^^,^^25^^,^^26^^,^^27^^,^^28^ The thoracic pleuron is strikingly different across these three main pterygote lineages and their stem groups, each exhibiting specific modifications, probably reflecting different requirements for flight.^2^^,^^5^^,^^24^^,^^28^ Besides these three crown groups and their stem groups, another significant Paleozoic superorder of pterygotes must be considered, namely, the Palaeodictyopterida.

This group occurred in the ecosystems of the Carboniferous and Permian, with its earliest record from the early Late Carboniferous, about 325 million years ago.^29^ Thus, they represented some of the earliest pterygotes and exhibited a spectacular range of wing designs and flight abilities that have no parallel in our modern fauna.^29^^,^^30^ The phylogenetic position of Palaeodictyopterida is disputed; they have been hypothesized to belong to the Palaeoptera,^31^ to be an ingroup of Neoptera,^32^ or the sister group of Neoptera.^33^^,^^34^ Nevertheless, even their position as the most basal lineage of Pterygota cannot be definitely excluded.^19^^,^^35^^,^^36^ Surprisingly, the detailed morphology of the thorax was only scarcely studied in Palaeodictyopterida. Most relevant works deal with the structure of the wing base or alinota,^31^^,^^37^^,^^38^^,^^39^^,^^40^^,^^41^^,^^42^ whereas the structure of the lateral part of the thorax (pleuron) is mostly unknown, despite their remarkable diversity and frequency in the fossil record.^43^ In this study, we examined the thorax of a small (wingspan approx. 30 mm) palaeodictyopteran, *Eubleptus danielsi* Handlirsch, 1906 (Eubleptidae) from sphero-siderite concretions of the Carboniferous (Moscovian) locality Mazon Creek Lagerstätte, situated in Illinois (USA).^44^^,^^45^ We present new material of *E. danielsi* and supplement the previous research on specimens described by Handlirsch,^46^ and Carpenter.^47^^,^^48^

## Results

The material studied herein consists of specimens with different aspects of preservation (both lateral and dorsal, see STAR Methods section), allowing us to both describe the thoracic structures in 3D for the first time and get a complex picture of the thorax. The aim is to present the structure of the pleuron in Palaeodictyopterida for the first time, to critically compare the morphology of the pleuron with the other main pterygote lineages, such as Odonatoptera, Panephemeroptera, and Neoptera, and to reconstruct the arrangement of the lateral thoracic body wall as it was at the beginning of pterygote evolution. Furthermore, the 3D reconstruction enables us to study the morphology in the context of flight-related traits. With due recognition of the limitations inherent in inferring performance from fossil morphology, these estimates allow us to formulate a hypothetical scenario concerning the flight capacity of *Eubleptus*.

**Figure 1:**
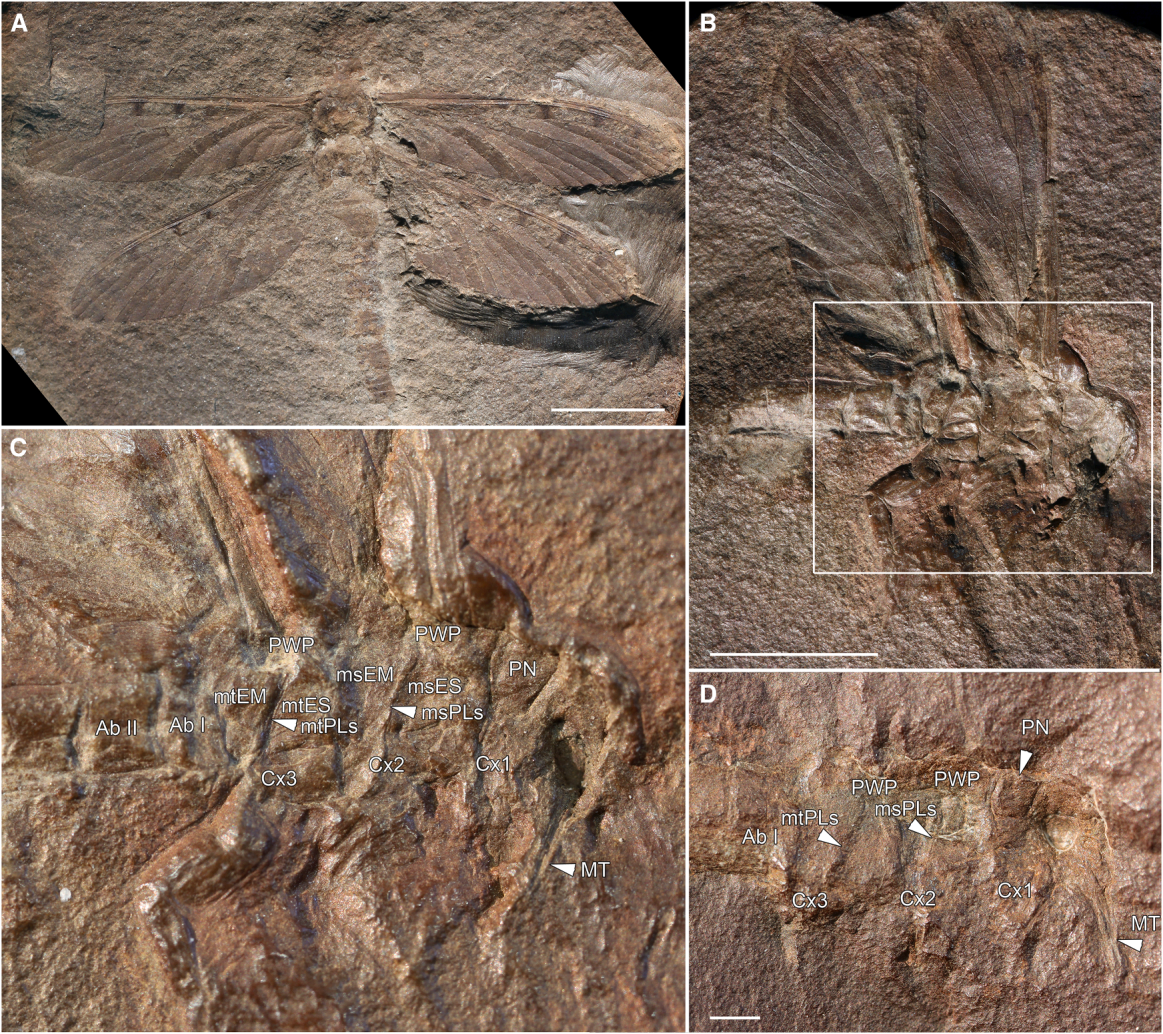
*Eubleptus danielsi*, palaeodictyopteran from Pennsylvanian of Mazon Creek (Illinois, USA) (A) Habitus in dorsal view with outstretched wings, FM PE 40223. Scale bar, 5 mm. (B and C) Habitus in lateral view with the upright position of wings at rest and detail of head, thoracic pleuron and proximal segments of abdomen, FM PE 32072. Scale bar, 5 mm. (D) Habitus in lateral view of head, thorax and basal part of abdomen, FM PE 22016. Scale bar, 1 mm. Abbreviations: Ab, abdominal segment; Cx, coxa; msPLs, mesopleural suture; msES, mesoepisternum; msEM, mesopimeron; mtPLs, metapleural suture; mtES, metaepisternum; mtEM, metaepimeron; PN, pronotum; MT, mouthparts in form of rostrum; PWP, pleural wing process.

In the material of *E. danielsi* we studied, the prothorax is markedly reduced in contrast to the meso- and metathorax (Figure 1), with the lobe of the pronotum (Pn) slightly expanded, with a prominent, acute posterolateral angle without venation or articulation, with the meso- and metathoracic wings held upright at rest position (like in extant mayflies). The pleural region is well sclerotized with discernible pleurites. The propleuron is anteriorly concealed by the lateral parts of the Pn, but clearly reduced, in contrast to the meso- and metapleuron (Figures 1B–1D).

The prominent feature of the pleuron of the meso- and metathorax in *E. danielsi* is the pleural suture (msPLs, mtPLs), which extends between the pleural wing process (PWP, providing a fulcrum for the wing) and the coxa (Cx). It divides a pleuron into an anterior episternum (msES, mtES) and a posterior epimeron (msEM, mtEM). The pleural suture is present in most extant pterygotes, produced internally as the pleural apophysis (also termed pleural ridge), which is usually locally extended into the pleural arm, connecting with the corresponding sternal apophysis via muscle fibers. This structure forms the internal skeletal reinforcement that strengthens the thoracic segment wall.^38^ The pleural ridge dorsally articulates with the second axillary sclerite of the wing base (2Ax, not preserved in *E. danielsi*, but it can be estimated based on the wing base orientation). Because Palaeodictyopterida are very rarely preserved in lateral view, there is limited evidence to determine whether this arrangement is universal across this entire group. However, the only other palaeodictyopteridan known from a lateral aspect, *Permuralia maculata* (order Diaphanopterodea), exhibits a similar arrangement of the pleural sutures, hinting toward universality.^39^

**Figure 2:**
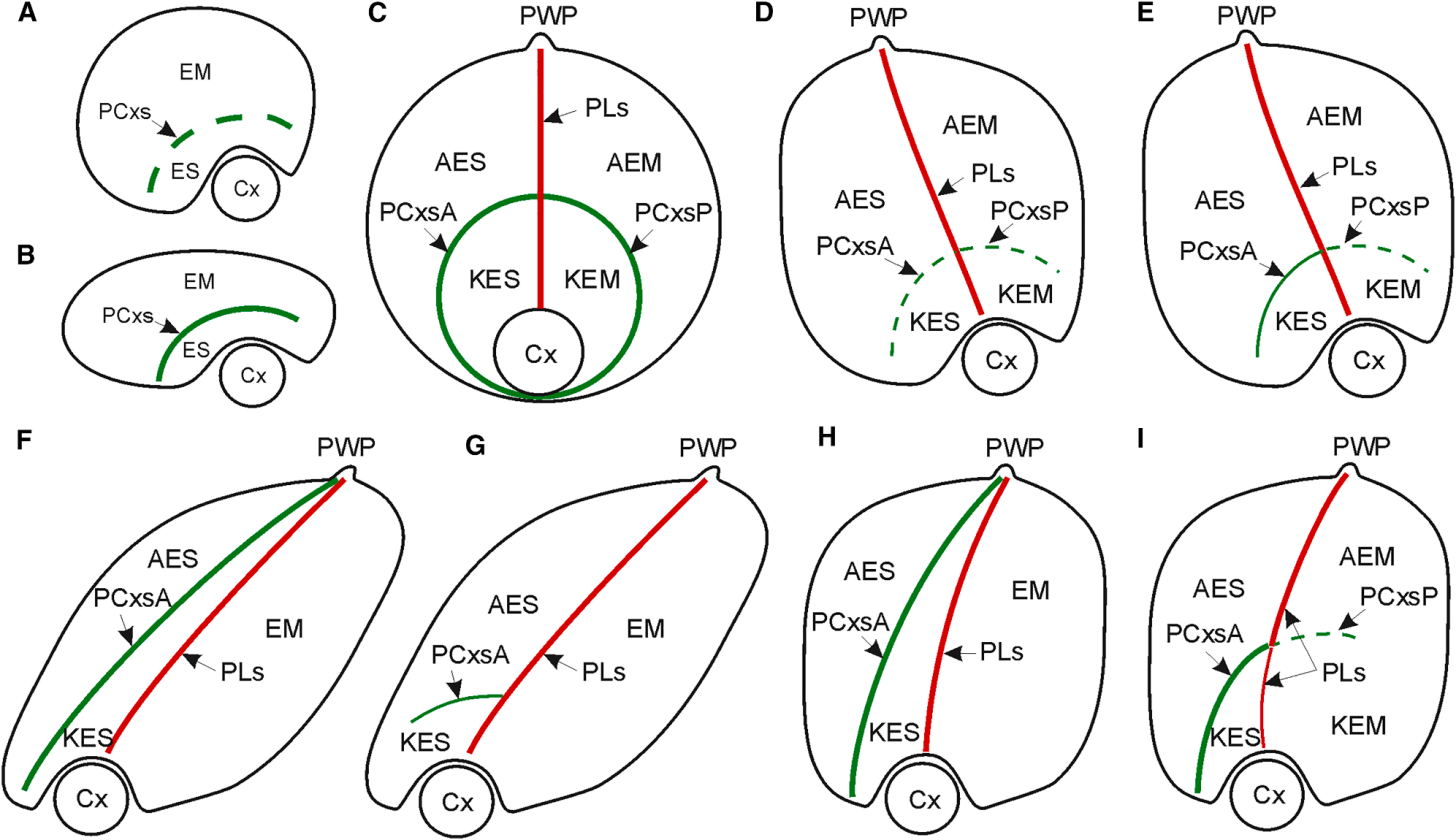
Schematic drawings of mesothoracic pleural structures in main groups of insects (A) Bristletails (Archaeognatha). (B) Silverfish (Zygentoma). (C) Simplified diagram of pterothoracic pleuron according to Matsuda (1970). (D) *Eubleptus danielsi* (Palaeodictyoptera). (E) Stoneflies (Neoptera). (F) Stem-odonates (Meganisoptera). (G) Crown-odonates (Odonata). (H) Stem-mayflies (Permoplectoptera). (I) Crown-mayflies (Ephemeroptera). Abbreviations: AES, anepisternum, AEM, anepimeron, Cx, coxa, EM, epimeron, KES, katepisternum, KEM, katepimeron, PWP, pleural wing process, PLs, pleural suture (in red), PCxsA, anterior paracoxal suture (in green), PCxsP, posterior paracoxal suture (in green). Pronounced suture indicated by a thicker line. Suture indistinct or missing in some taxa indicated by dashed line. Other elements present in pleura, but not recognizable in *E. danielsi* and not discussed in the present study, are omitted in the drawings for clarity.

**Figure 3:**
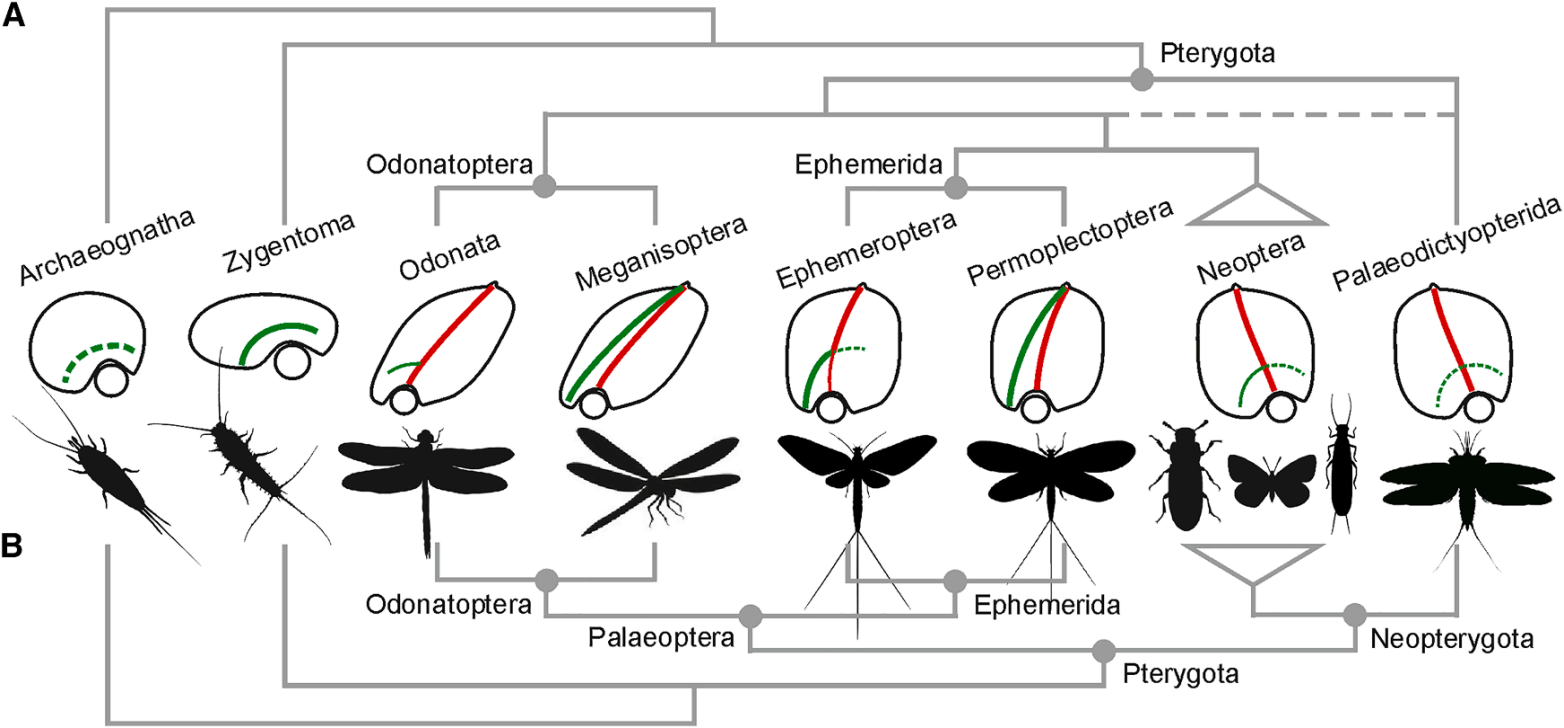
Two major scenarios of evolutionary relationships in insects with schematic structure of pleuron (A) Chiastomyaria hypothesis as a plausible alternative to Palaeoptera after Simon et al.^28^ The position of Palaeodictyopterida close to the basal divergence of pterygotes. (B) Palaeoptera hypothesis with Palaeodictyopterida as a sister group of Neoptera sensu Misof et al.^12^ and Sroka et al.^34^ Paracoxal suture in green, pleural suture in red.

The pleural suture runs obliquely in *E. danielsi*, being inclined anteriorly in its dorsal portion (Figure 1D). When we compare this pattern with the major pterygote lineages (Figures 2 and 3), mayflies (Ephemeroptera, including their stem-group representatives of Permoplectoptera) exhibit the pleural suture slightly inclined in the opposite direction.^5^^,^^24^^,^^49^ Such an opposed inclination is even more extreme in Odonata, including its stem group Meganisoptera,^23^ where it relates to the apomorphically skewed synthorax of this order, likely reflecting an adaptation to aerial prey capture using the legs.^10^ Within Neoptera, the most common arrangement of the pleural suture is a tendency to run obliquely forward in its dorsal portion, although such a condition is not present universally and the suture may even be entirely absent, e.g., in Heteroptera.^2^ As hypothesized by Matsuda,^2^ the course of this suture was probably nearly vertical in ancestral Pterygota (see Figure 2). As demonstrated above, the inclination varies between major pterygote lineages, and which pattern is closest to the pterygote ground plan (the set of all plesiomorphic and autapomorphic characters, see Beutel et al.^50^) is not straightforward to establish (Figure 3). Nevertheless, the direction of the pleural suture in *E. danielsi* is very similar to the most common situation in neopterous insects. When considering the ancestral position of the pleural suture in Pterygota, we should take into account the lepismatid organization of the pleuron,^9^^,^^10^^,^^51^ i.e., the position of the coxae, subcoxal sclerites and paranotal lobes in silverfish (Zygentoma), which represents the extant sister group to winged insects.^12^ The pleural suture is absent in silverfish.^2^ However, the coxae are situated relatively posteriorly on their respective thoracic segments, and the axis between the coxae and corresponding paratergites with hypothetical position of wing pivoting point are not vertical, but clearly inclined like in *E. danielsi* (see Figure 2). Hence, this might represent the initial direction of the pleural suture for ancestral pterygotes.

Another characteristic of the pleuron in *E. danielsi* is the lack of a clearly distinguishable paracoxal suture. Kobayashi^52^^,^^53^ demonstrated in an embryological study that the paracoxal suture separates subcoxa1 and subcoxa2, which correspond to the anapleural and katapleural rings. Thus, the paracoxal suture originally led perpendicularly to the pleural suture and was present in both the episternum and epimeron, as seen in apterygote orders, whereas they are retained only in the episternum as the anterior paracoxal suture in most pterygotes^2^. Therefore, some degree of reduction of the posterior paracoxal suture on the epimeron probably occurred rather early in pterygote evolution.

The anterior paracoxal suture has been retained in most pterygotes, but subjected to changes in its direction (Figure 2). In stem-mayflies, the anterior paracoxal suture runs almost parallel with the pleural suture; these two sutures being connected on the dorsal part of pleuron near pleural wing process.^24^ A similar arrangement is reported for the stem-odonates of the family Meganeuridae (Rasnitsyn and Pritykina^23^: fig. 99). Such a derived course of the anterior paracoxal suture is interestingly not shared in the crown groups of both orders. In crown-group odonates, the pleural and paracoxal sutures meet at a wide angle at about 1/4 of the former.^23^ In crown-group mayflies, the anterior paracoxal suture connects to the pleural suture approximately in the middle of its length, and continues as a faint posterior paracoxal suture on the epimeron.^5^^,^^49^ Where both pleural and paracoxal sutures are retained within Pterygota, the pleural suture is usually more prominent. In stem-mayflies and stem-odonates, anterior paracoxal and pleural sutures are comparable in thickness. It is only in the crown-group mayflies that the anterior paracoxal suture is more prominent when compared with the pleural suture. Such a strengthening of the anterior paracoxal suture represents an apomorphy of crown-group mayflies.

We did not observe an unambiguous paracoxal suture in *E. danielsi*. Even if this suture was present and just not visible in the fossil, it was certainly rather faint and not as prominent as in crown-mayflies, nor of a comparable thickness to the pleural suture as in stem-mayflies and stem-odonates. Both of these arrangements are derived, so *E. danielsi* might have represented a situation more similar to the pterygote ground plan. The general arrangement of the pleural wall of the pterothorax in *E. danielsi* (prominent oblique anteriorly inclined pleural suture, without distinct paracoxal suture) resembles that of neopterous insects, rather than mayflies or odonates, or their stem-group representatives.

## Discussion

### Flight-related traits and wing resting position in *E. danielsi*

**Figure 4:**
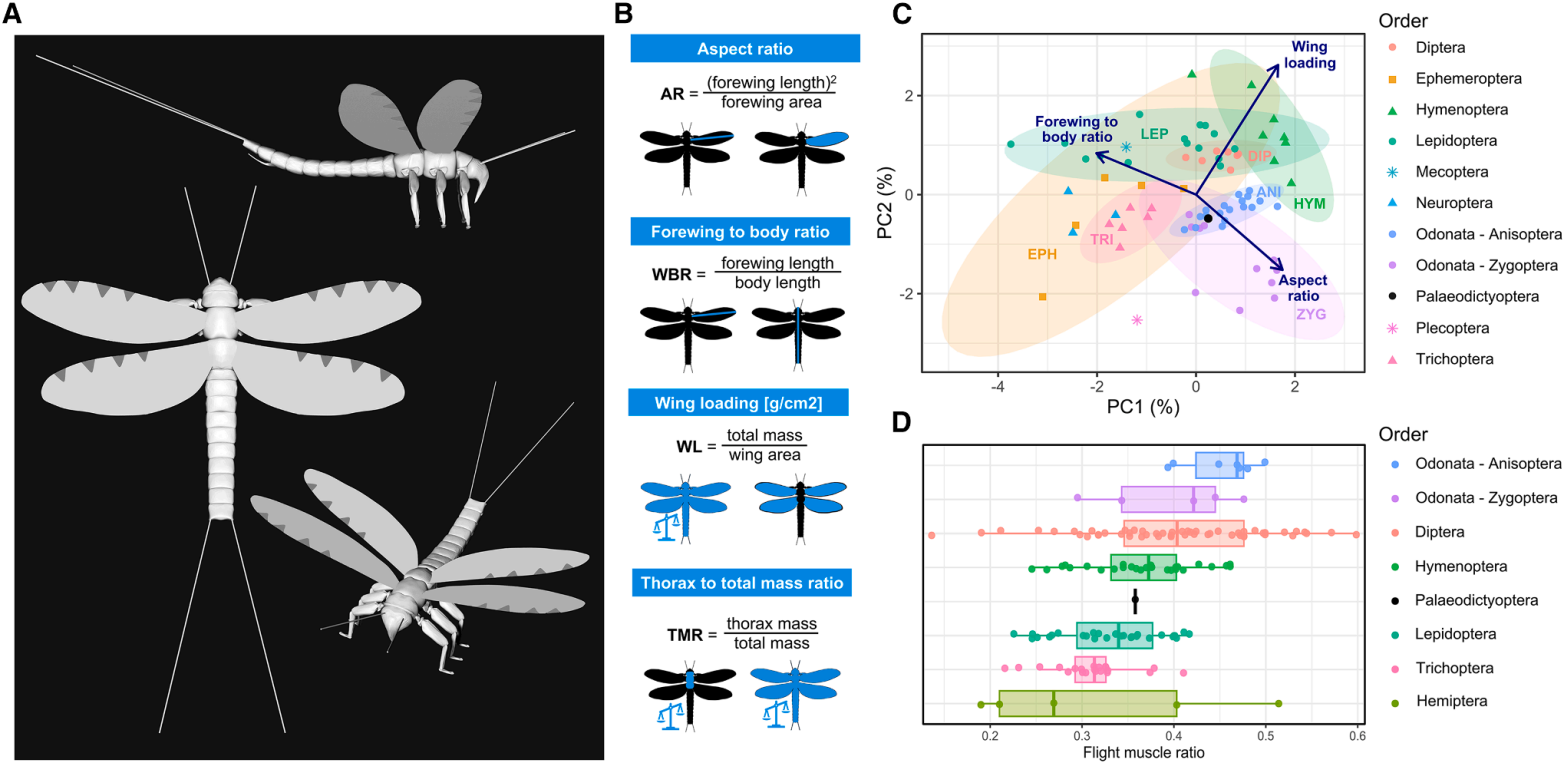
Flight-related traits of *Eubleptus danielsi* and recent insect species (A) 3D model of *Eubleptus danielsi*. (B) Flight-related morphological traits used in the analyses. (C) Principal component analysis (PCA) of wing morphology of extant insect species and *Eubleptus danielsi*, based on forewing to body ratio, aspect ratio and wing loading. (D) Flight muscle ratio (FMR) of extant insects and *Eubleptus danielsi*.

To explore how the flight-related morphology of *Eubleptus danielsi* compares with that of extant insects, we focused on a set of anatomical proxies that are relevant to flight, quantifiable across a broad taxonomic range, and inferable from fossil morphology.^30^^,^^54^^,^^55^^,^^56^^,^^57^^,^^58^ These included wing loading, calculated as body mass divided by total wing area; wing-to-body ratio, expressing relative wing length; wing aspect ratio, describing the relative elongation of the wings; and flight muscle ratio (FMR), calculated as flight muscle mass divided by total body mass. Based on specimens of *E. danielsi* that preserve both lateral and dorsal aspects, we reconstructed a 3D model (see Figure 4A). Using this model and a mean insect body density known from the extant insects,^59^ we estimated its wing loading, aspect ratio, wing-to-body ratio, and FMR (Figure 4B). We then carried out an exploratory principal component analysis including *E. danielsi* and extant representatives of several insect orders (Figure 4C). In this analysis, *E. danielsi* plotted close to groups with homonomous wings, which are characterized by moderate values of wing loading, wing-to-body ratio, and aspect ratio. Its nearest neighbors in the morphospace were Palaeoptera, especially small Anisoptera and narrow-winged Zygoptera. However, *E. danielsi* exhibits several differences in thoracic morphology in comparison to them, such as a presumably lower FMR (Figure 4D). FMR (calculated as flight muscle mass/total mass) is a strong determinant of maximum lift production^54^ and short-duration flight performance in extant insects^56^ (Berwaerts et al. 2002). Although the relationship between FMR and flight speed or overall flight style is more complicated and context-specific, affected by e.g., muscle physiology and wing kinematics,^55^^,^^60^^,^^61^^,^^62^^,^^63^ the low FMR suggests that *E. danielsi* had a more limited capacity for forceful, high-power flight. This is further supported by *E. danielsi’s* strongly corrugated wings and elongated cerci, which would be expected to increase drag and thus reduce flight speed, pointing to slower but stable flight.^64^^,^^65^^,^^66^ The cerci probably functioned analogously to those of Ephemeroptera, stabilizing the flight and allowing it to alternate between a controlled descent with spread cerci and active flapping.^10^^,^^66^ Among the Permian insects, we can find possible parallels in the flight style of *E. danielsi* and the stem-mayflies (Permoplectoptera) with homonomous wings, and possibly also the palaeodictyopteran family Dictyoneuridae, as suggested by Wootton and Kukalová-Peck.^30^

*Eubleptus* was unique among Palaeodictyoptera in its apparent ability to hold the wings vertically at rest, somewhat reminiscent of mayflies and damselflies. This wing posture does not represent an artifact of preservation, since we report three specimens with the same position of the wings, resembling, e.g., fossils of Permian stem-mayflies. In damselflies, holding the wings together has been suggested to reduce the visibility of light reflected from the wing membranes, potentially lowering predation risk.^67^ Given that *E. danielsi* is among the smallest Palaeodictyoptera known to date (some representatives of this order reached a wingspan up to 55 cm), it may likewise have been more vulnerable to predators, and vertical wing positioning could have functioned as an antipredator adaptation. This posture might also have reduced the likelihood of wing damage or wetting in moist habitats. Nevertheless, wing positioning is primarily determined by the configuration of the wing base and associated musculature rather than by the pleuron itself. Indeed, although the arrangement of the pleural sutures in *Eubleptus* resembles that seen in Neoptera, the wing resting position is different, as *Eubleptus* could not fold its wings flatly on the abdomen like many Neoptera do today.

### Outlook

The uniquely preserved pleuron of *Eubleptus danielsi* brings new insight into the early evolution of insect wings and tackles the unresolved issue of the basal split of Pterygota. The pleuron in *E. danielsi* is comparable in some respects to Neoptera in the arrangement of pleural and paracoxal sutures. However, the same arrangement can be the consequence of the partial flatness of thoracic segments, combined with the posterior location of the coxae, as is present in silverfish (Zygentoma), the sister group of all pterygote insects.

Thus, the pleuron of the protopterygote, the hypothetical first flying insect, would likely be more similar to *E. danielsi* than to the arrangement in other basal pterygotes, such as mayflies or dragonflies and damselflies. The flight-related traits of *E. danielsi* as reconstructed herein suggest some similarities with small Odonata, albeit with presumably lower FMR, which, together with long cerci and small body size, hints toward a less powered flight mode. Our results provide a new, complex insight into how the thoracic structure and flight-related characteristics may have looked at the very beginning of insect flight evolution.

### Limitations of the study

This study is based exclusively on well-preserved fossil specimens of the palaeodictyopteran *Eubleptus danielsi*. Uniquely, these specimens show the thoracic body wall—the pleuron. However, for further discussion of flight abilities, more complete preservation of the flight apparatus, including the wing base, would be essential. Unfortunately, these structures consist of sclerites and membranes which have a low potential for preservation, due to weak sclerotization. A further limitation of this study in the reconstruction of the protopterygote is that the current estimations of pterygote divergence, based on molecular clock analyses, indicate that divergence occurred in the Lower Devonian, about 400–405 mya, while the first undisputed fossil evidence of Pterygota comes from the mid-Carboniferous (Mississippian, ca 324 mya).

## Resource availability

### Lead contact

Further information and requests for resources should be directed to and will be fulfilled by the lead contact, Jakub Prokop (jprokop@natur.cuni.cz).

### Materials availability

All included specimens of palaeodictyopteran *Eubleptus danielsi* are accessioned in The Field Museum of Natural History (Chicago, U.S.A.) under the accession nos. (FM PE40223, FM PE32072, and FM PE22016).

### Data and code availability

• All data and code reported in this paper can be found in the supplementary (Data S1. Dataset of flight-related traits of recent insect species and Eubleptus danielsi, Data S2. R script used for conducting PCA and generating plots, Data S3. 3D model of Eubleptus danielsi) and are publicly available as of the date of publication.
• Any additional information required to reanalyze the data reported in this paper is available from the lead contact upon request.

## Acknowledgments

The authors are grateful to Paul Mayer (The Field Museum, Chicago, USA), who kindly provided access to the collection under his care. K.D. thanks to Anna Kubicová for providing samples of dragonflies for cross-checking of the FMR data. All authors acknowledge financial support from the 10.13039/501100001824Czech Science Foundation (grant no. 24-11498S).

## Author contributions

J.P. and P.S. conceived the initial idea and designed the project. J.P., K.D., L.S., and P.S. examined material by light stereomicroscopy, analyzed the data, discussed the results, and wrote the paper. K.D. designed the flight-related trait analysis, gathered and calculated the data, performed principal component analysis, created a 3D virtual model, and interpreted the flight-related traits of *E. danielsi*. J.P., K.D., and P.S. designed the figure plates.

## Declaration of interests

The authors declare no competing interests.

## STAR★Methods

### Key resources table

**Table:** 

REAGENT or RESOURCE	SOURCE	IDENTIFIER
**Deposited data**		

Dataset of flight-related traits of extant insect species and *Eubleptus danielsi*	This study and references listed in Data_S1	Data_S1_Eubleptus_dataset.csv
R script used for conducting PCA and generating plots	This study	Data_S2_Eubleptus_code.R
3D model of *Eubleptus danielsi*	This study	Data_S3_Eubleptus_3Dmodel.blend

**Software and algorithms**		

Adobe Photoshop CS6	Adobe Systems	https://www.adobe.com/products/photoshop.html
Helicon focus Pro 6.2.2	Helicon Soft Ltd	https://www.heliconsoft.com/heliconsoft-products/helicon-focus/
Zerene Stacker	Zerene Systems	https://www.zerenesystems.com/cms/stacker
Inkscape 1.2.2	InkScape	https://www.inkscape.org/
Blender 4.0	Blender Foundation	https://www.blender.org/download/releases/4-0/
Microsoft PowerPoint 365	Microsoft Corporation	https://www.microsoft.com/cs-cz/microsoft-365
ChatGPT 5.0	OpenAI	www.chatgpt.com
R 4.2.2	The R Foundation for Statistical Computing	https://cran.r-project.org/
RStudio 2026.01.1	Posit Software, PBC	https://posit.co/download/rstudio-desktop/
ImageJ	Schneider et al. 2012	https://imagej.nih.gov/ij/

### Experimental model and subject participant details

#### Specimen repositories

The specimens of *Eubleptus danielsi* in this study were examined from the following institutional collection: FM, The Field Museum of Natural History (Chicago, U.S.A.).

### Method details

#### Specimen imaging and optical devices

The present results were obtained by comparative morphological study using optical stereomicroscopy. Observations were performed using a Zeiss Discovery V20. Photographs were taken with a Canon D550 (Canon Inc., Tokyo, Japan) digital camera, with MP-E 65mm and EF 50mm lenses. The original photographs were processed using Adobe Photoshop CS6 (Adobe Systems, San Jose, California, U.S.A.), and for some images the focus-stacking software Helicon focus Pro 6.2.2 (Helicon Soft Ltd., Kharkov, Ukraine) or Zerene Stacker (Zerene systems LLC, Richland, U.S.A.) were used.

### Quantification and statistical analysis

To study the flight-related morphology of *Eubleptus danielsi* in comparison with extant insects, we compiled a dataset spanning multiple insect orders and including four flight-related metrics: wing-to-body ratio, wing aspect ratio, wing loading, and flight muscle ratio (FMR). These traits were selected because they allow comparisons across insects that differ substantially in body size, can play a role in the flight performance, and have been widely measured in previous studies across multiple insect orders, making them available for broad comparative analysis. We measured forewing length, body length, forewing area, and total wing area of *Eubleptus danielsi* from photographs using ImageJ, and constructed a 3D model of *E. danielsi* in Blender to estimate total body and thoracic volumes. During the process, all dimensions were cross-checked between laterally and dorsally preserved fossil specimens to construct as precise 3D model as possible. Using these volumes and an assumed insect density of 0.65 g/cm^3^ after Drake et al.,^59^ we calculated its body mass and flight muscle mass, the latter estimated as 95% of thorax mass, and calculated flight muscle ratio (FMR) as flight muscle mass/total mass. We also compiled forewing length, body length, body mass, forewing area, and total wing area for 77 extant insect species from 10 orders and FMR for 146 extant insect species from 7 orders based on the published literature. Where FMR was unavailable but thorax mass was reported, flight muscle mass was estimated as 95% of thorax mass and FMR was calculated as flight muscle mass divided by total body mass. For species with missing wing and body lengths or areas, we measured them manually from images using ImageJ. We calculated the forewing-to-body ratio as forewing length divided by body length, and the forewing aspect ratio as forewing length squared divided by forewing area, and the wing loading as body mass divided by total wing area. The full dataset, analysis script, and references for all source articles, photographs and 3D model are provided in the supplemental information (Data S1: dataset and references, Data S2: script, Data S3: 3D model).

We performed principal component analysis (PCA) in R, using wing loading, wing to body ratio and aspect ratio for each species. Wing loading was log-transformed and standardized (z-scores), while forewing-to-body ratio and forewing aspect ratio were standardized to zero mean and unit variance. PCA was conducted using the princomp function on the standardized trait matrix, including *Eubleptus danielsi*, whose position in morphospace was visualized relative to extant insect orders. PCA loadings were extracted and rescaled for visualization as vectors in the ordination space. Morphological disparity among insect orders was illustrated by plotting species scores on the first two principal components and drawing 95% confidence ellipses for orders represented by at least five species.

## Supplemental information

Data S1. Dataset of flight-related traits of recent insect species and *Eubleptus danielsi*

Data S2. R script used for conducting PCA and generating plots

Data S3. 3D model of *Eubleptus danielsi*
